# Hearing Protection Among Workers Exposed to Occupational Noise in the South African Aluminium Industry

**DOI:** 10.3390/ijerph23030306

**Published:** 2026-02-28

**Authors:** Nomfundo Moroe, Asibonge Shandu

**Affiliations:** Speech Pathology and Audiology Department, University of the Witwatersrand, Johannesburg 2001, South Africa; 2590508@students.wits.ac.za

**Keywords:** occupational noise, hearing protection devices, noise-induced hearing loss, South Africa, worker behaviour

## Abstract

**Highlights:**

**Public health relevance—How does this work relate to a public health issue?**

- Occupational noise-induced hearing loss remains a prevalent, preventable hazard affecting workers’ hearing and overall well-being.
- Understanding real-world patterns of hearing protection device (HPD) use identifies gaps between awareness, access, and consistent protective behaviour in high-noise industries.

**Public health significance—Why is this work of significance to public health?**

- Provides empirical evidence on the determinants of HPD use in the under-researched aluminium manufacturing sector, complementing mining-focused studies.
- Highlights how demographic, device-related, and organisational factors influence protective behaviour and ONIHL risk.

**Public health implications—What are the key implications or messages for practitioners, policy makers and/or researchers in public health?**

- Effective hearing conservation programmes require multi-level strategies, including appropriate HPD selection, tailored training, and reinforcement of workplace safety culture.
- Monitoring, feedback, and context-specific interventions are critical to improving HPD compliance and reducing occupational hearing loss.

**Abstract:**

Background: Occupational noise-induced hearing loss (ONIHL) remains one of the most prevalent occupational diseases globally and in South Africa. Despite awareness and regulatory frameworks, consistent use of hearing protection devices (HPDs) is suboptimal in high-noise industries. Aim: To investigate patterns of HPD use and the factors influencing compliance among workers in an aluminium manufacturing company exposed to noise levels exceeding 85 dB(A). Methods: A cross-sectional survey was conducted with 115 workers, including 68.7% males and 31.3% females. Chi-square tests assessed associations between categorical variables, and logistic regression identified significant predictors of consistent HPD use. Results: Although 94.8% of workers were aware of the risks of hazardous noise, only 51.3% reported always using HPDs. Gender, education level, type of HPD, type and duration of noise exposure, and perceived susceptibility to hearing loss were significantly associated with consistent HPD use. Logistic regression revealed that gender, type of HPD, type of noise exposure, and perceived susceptibility significantly predicted consistent use. Conclusions: Despite high awareness and access to HPDs, consistent use remains moderate and is influenced by demographic, perceptual, device-related, and workplace factors. Findings highlight the need for targeted interventions, training, and workplace strategies to improve HPD compliance and prevent ONIHL.

## 1. Introduction

Noise is defined as unwanted sound produced by humans or machines [1]. Exposure to noise can impair hearing and induce a range of physiological, psychological, and pathological effects. Beyond auditory impacts, noise disrupts essential human activities, including work performance, rest, sleep, and communication [2], making occupational noise a significant public health and workplace safety concern.

Globally, over 1.5 billion people live with some degree of hearing loss, and by 2050, more than 700 million are projected to experience disabling hearing loss [3]. Occupational noise-induced hearing loss (ONIHL) is one of the most common occupational diseases worldwide [4]. Although national prevalence data for South Africa are lacking, global estimates suggest that about 16% of permanent adult hearing loss is attributable to occupational noise exposure [5]. ONIHL negatively affects workers’ well-being, causing auditory issues such as tinnitus and threshold shifts, as well as non-auditory effects including stress, fatigue, headaches, and sleep disturbances [1,6,7]. It is the leading occupational disability globally [8] and the second most common cause of acquired hearing loss after presbycusis [9], with social and economic consequences such as communication difficulties, reduced productivity, and increased risk of job loss [10].

Although noise exposure is prevalent across industries [11], South African research on ONIHL has primarily focused on mining [12,13,14], with less attention paid to high-risk manufacturing sectors such as aluminium production. In aluminium manufacturing, noise from power tools, heavy machinery, and metal processing often exceeds permissible exposure limits, e.g., smelting operations produce 100–120 dBA compared to the recommended 85 dBA over eight hours [4,15]. Consequently, ONIHL remains a significant hazard in this sector, contributing to communication difficulties, reduced quality of life, and stress [4].

International and South African regulations mandate limits of 85 dBA averaged over an eight-hour workday and require employers to implement noise control measures, provide hearing protection, conduct audiometric surveillance, and offer training [16,17]. ONIHL is also compensable under South African law, reinforcing the importance of accurate exposure records and employee compliance. Despite these frameworks, ONIHL persists, indicating limitations in the implementation or effectiveness of current preventive strategies.

Although irreversible, ONIHL is preventable through effective Hearing Conservation Programmes (HCPs) [18], which include noise measurement, engineering and administrative controls, personal hearing protection, audiometric monitoring, and training [12]. Engineering controls are preferred, but practical challenges often leave hearing protection devices as the main protective measure [19,20]. Their effectiveness depends on proper selection, fit, consistent use, and maintenance [21], and evidence shows that correct, continuous use reduces the risk of ONIHL [22,23,24]. Nonetheless, non-compliance remains common [25], influenced by discomfort, communication difficulties, workplace culture, and workers’ risk perceptions [24,26,27,28,29]. Understanding the factors that influence HPD use is therefore critical to designing interventions that promote consistent adherence and reduce the burden of occupational hearing loss.

Given this context, the present study aimed to examine factors associated with HPD use among workers in a South African aluminium manufacturing setting. The specific objectives were as follows:

1. To describe the frequency of HPD use among workers.
2. To determine whether HPD use frequency differs according to demographic characteristics.
3. To assess the influence of perceptual factors, including perceived risk of hearing loss, confidence in correct HPD use, and perceived discomfort, on HPD use frequency.
4. To examine the association between type of HPD, type of noise, and duration of noise exposure with consistent HPD use.
5. To evaluate the impact of workplace factors, including training, peer and supervisor influence, and safety culture, on consistent HPD use.

Based on these objectives and existing literature, the following hypotheses were formulated:

**H1.**  *The frequency of HPD use varies among workers.*

**H2.**  *HPD use frequency differs by demographic characteristics.*

**H3.**  *Perceptual factors (perceived risk of hearing loss, confidence, and discomfort) are associated with HPD use frequency.*

**H4.**  *The type of HPD, type of noise, and duration of exposure influence consistent HPD use.*

**H5.**  *Workplace factors (training, peer and supervisor influence, and safety culture) impact consistent HPD use.*

In South Africa, research on HCPs outside the mining sector, for example, in aluminium and other manufacturing industries, is limited [10,11,12]. Few studies have examined how demographic, perceptual, device, and workplace factors interact to determine HPD adherence. By addressing this gap, the present study provides evidence to guide the development of contextually relevant interventions aimed at improving protective behaviour. The findings are expected to inform practical strategies, including tailored training programs, HPD selection and fit strategies, and organizational policies, thereby enhancing hearing conservation and reducing the burden of occupational hearing loss.

## 2. Materials and Methods

### 2.1. Study Design

This study employed a cross-sectional design [30] to examine factors associated with the frequency of hearing protection device (HPD) use among workers exposed to occupational noise levels exceeding 85 dB(A). Data were collected using a structured quantitative survey that captured information on demographic characteristics, perceptual factors (such as perceived risk of hearing loss, confidence in correct HPD use, and perceived discomfort), device-related factors (the type of HPD), noise exposure characteristics (type and duration), and workplace influences (training, peer and supervisor support and safety culture). This design allowed for the identification of patterns of HPD use and the associations between individual, device, and organizational factors within a specific industrial context.

### 2.2. Study Setting

This study was conducted at an aluminium manufacturing company located in Gauteng, South Africa. The company is the leading aluminium extruding supplier in Africa and represents a large-scale manufacturing environment encompassing metal smelting, extrusion, and machining operations. The site was selected due to the presence of sustained high-noise work environments that place workers at risk for ONIHL. During a third-year practicum in Occupational Audiology, the primary researcher observed inconsistent use of HPDs among workers exposed to hazardous noise, despite the availability of protective devices. This observation highlighted a gap between HPD provision and consistent use, which informed the rationale for this study. The workforce at the site is diverse with respect to age, gender, education level, and job roles, allowing for the examination of how demographic and workplace factors influence HPD use behaviours.

### 2.3. Study Population and Sampling

The study population comprised workers employed at the aluminium manufacturing company who were routinely exposed to noise levels exceeding 85 dB(A). Participants performed a range of occupational roles, including machine operators, production line workers, and maintenance personnel, all of which involved direct exposure to high levels of occupational noise. The company employs approximately 600 staff members, including factory-based and office-based employees. Office-based employees are not routinely exposed to hazardous noise and are therefore not required to use HPDs. As this was an exploratory study, no formal sample size calculation was undertaken. A total of 115 participants completed the survey, representing workers who met the inclusion criteria and were available during the data collection period. Purposive sampling was used to recruit participants who met the inclusion criteria [31]. This sampling strategy ensured inclusion of workers with direct experience of occupational noise exposure and HPD use, thereby enabling the focused examination of factors influencing HPD compliance. Inclusion criteria were employees exposed to hazardous noise levels of ≥85 dB(A), the provision of HPDs by the employer, aged 18 years or older, and willingness to participate. Exclusion criteria included office-based workers not routinely exposed to hazardous noise and employees exposed to noise levels below 85 dB(A).

### 2.4. Data Collection Instrument

Data were collected using a self-developed questionnaire informed by existing literature [32,33,34]. The questionnaire was administered electronically using REDCap (version 16.1.3 Vanderbilt University 2026, Nashville, TN, USA) and consisted of 58 items addressing demographic characteristics, attitudes and beliefs regarding hearing protection, and perceived barriers and facilitators to HPD use. The survey was available in English and required approximately 15–20 min to complete. REDCap was selected as the data collection platform due to its secure, user-friendly interface and ability to facilitate confidential self-administration of surveys on participants’ personal devices. The platform also enabled the direct export of data into statistical software for analysis. A pilot study was conducted with two participants who met the inclusion criteria to assess clarity, terminology, and completion time. The pilot survey took approximately 12–15 min to complete, and no modifications were required based on the pilot findings [35].

### 2.5. Data Collection Procedure

Ethical clearance was obtained from the University Human Research Ethics Committee (non-medical) (Protocol number: STA_2025_41). Permission to conduct this study was obtained from the company, and a Non-Disclosure Agreement was signed. The researcher was allocated a designated space on site, and employees were informed about this study. Interested participants were directed to the survey area, where they reviewed the Participant Information Sheet and provided informed consent prior to participation. Participants completed the survey using either personal devices or devices provided by the researcher. Refreshments were offered as a token of appreciation for participation.

### 2.6. Ethical Considerations

Ethical principles of respect for autonomy, informed consent, confidentiality, and anonymity were adhered to throughout this study. Participants were informed of the study purpose, procedures, potential risks and benefits, and their right to withdraw at any time without consequence. Data collected via REDCap were stored securely and anonymised prior to analysis.

### 2.7. Data Analysis

Quantitative data were analysed using descriptive and inferential statistical methods [36]. Descriptive statistics were used to summarise participant characteristics and responses, with results presented as frequencies and percentages. Inferential analysis was conducted using the Chi-square test of independence to examine associations between demographic and workplace variables and HPD use.

## 3. Results

### 3.1. Summary of Participants’ Demographics

A total of 121 participants responded to the survey. Six participants did not complete all the questions, consequently, they were excluded from the analysis, resulting in a final sample size of 115 participants (Table 1).

Of the final sample, 76 participants (66.1%) identified as male, 38 (33%) as female, and one participant (0.9%) self-identified. Most participants were aged 45–64 years (50.4%), followed by those aged 25–44 years (47.8%). Only one participant fell into each of the 18–24 years and ≥65 years age categories. Regarding educational attainment, most participants had completed secondary education (65.2%), followed by tertiary education (31.3%), with smaller proportions reporting postgraduate (1.7%) or primary education (1.7%).

Work experience in noisy environments was predominantly long term. More than ten years of exposure was reported by 62.6% of participants, followed by 4–6 years (17.4%), 2–3 years (10.4%), 7–9 years (7%), and less than one year (2.6%). Most participants worked rotating shifts (67.8%), whereas 30.4% worked regular day shifts and 1.7% worked night shifts. Exposure to machinery noise was most reported (85.2%), followed by continuous background noise (7%), impact noise such as hammering (6.1%), and other noise types (1.7%).

### 3.2. Hearing Protection Device Use

**Figure 1:**
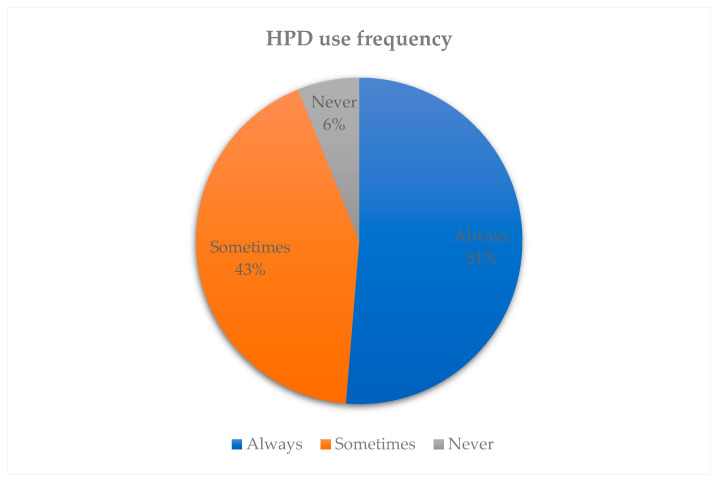
Frequency of HPD use.

Frequency of HPD use was categorised as “Always”, “Sometimes,” and “Never” (Figure 1). Among participants, 51% reported always using HPDs, 43% reported sometimes using HPDs, and 6% reported never using HPDs.

**Figure 2:**
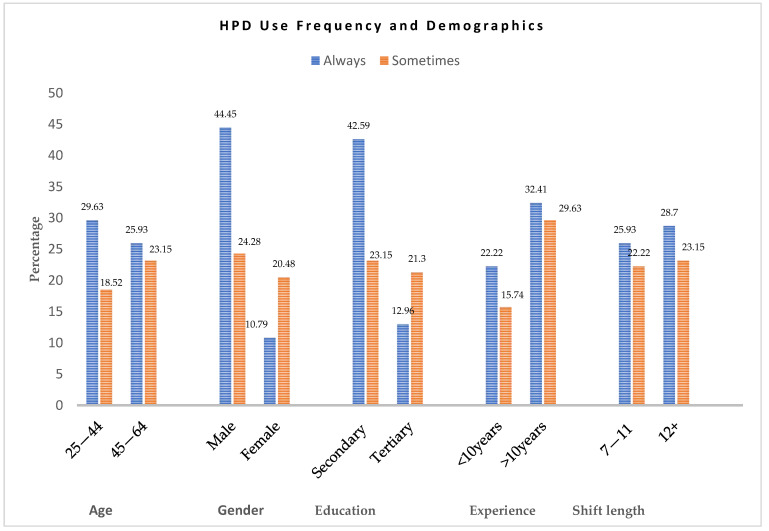
Comparison between consistent and inconsistent HPD use.

Participant characteristic data were further analysed according to the frequency of HPD use to identify any differences or similarities in their responses (Figure 2). Workers aged 25–44 were more likely to consistently use HPDs (29.6%) compared to occasional use (18.5%), whereas the 45–64 age group exhibited a smaller difference (25.9% always vs. 23.2% sometimes), suggesting less consistency among older workers. Gender disparities were evident: males reported the highest consistent use (44.4%) compared to 24.1% sometimes, whereas females had lower adherence (10.2% always vs. 20.4% sometimes). Education also influenced behaviour: participants with secondary education showed strong compliance (42.6% always vs. 23.2% sometimes), whereas tertiary-educated workers had lower consistency (13% always vs. 21.3% sometimes).

Work experience and shift duration further impacted HPD use. Employees with more than 10 years of experience reported the highest consistent use (32.41%) and relatively high occasional use (29.6%), whereas those with less than 10 years of experience showed lower adherence (22.2% always vs. 15.7% sometimes). Longer working hours were associated with higher HPD use: individuals working 12 or more hours exhibited stronger consistent use (28.7%) compared to 23.2% sometimes, whereas those working 7–11 h showed slightly lower consistency (25.9% always vs. 22.2% sometimes).

#### 3.2.1. Type of HPD Used

**Figure 3:**
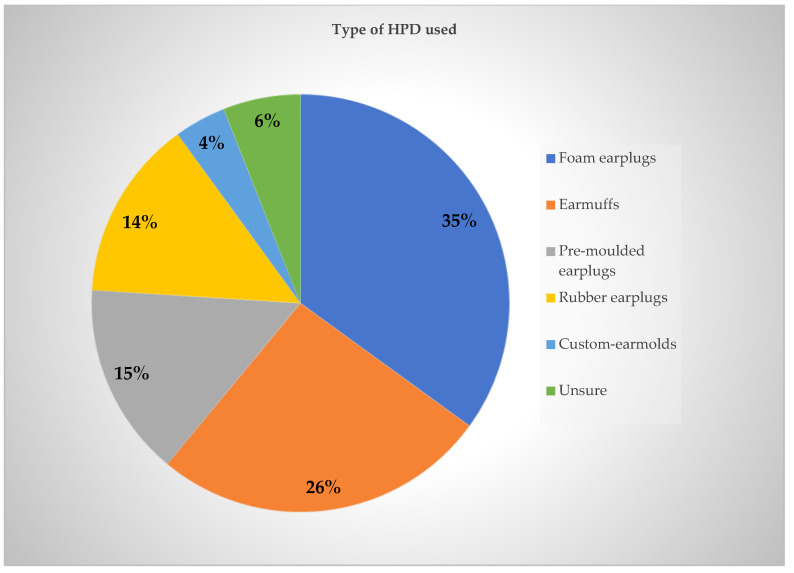
Types of HPD used.

When comparing workers who reported consistent use of hearing protection devices with those who reported inconsistent use, foam earplugs were the most reported type of HPD (35%), followed by earmuffs (26%), pre-moulded reusable earplugs (15%), rubber earplugs (14%), and custom-earmolds (4%). Six percent of participants were unsure of the type of HPD they used (Figure 3).

#### 3.2.2. Barriers to HPD Use

**Figure 4:**
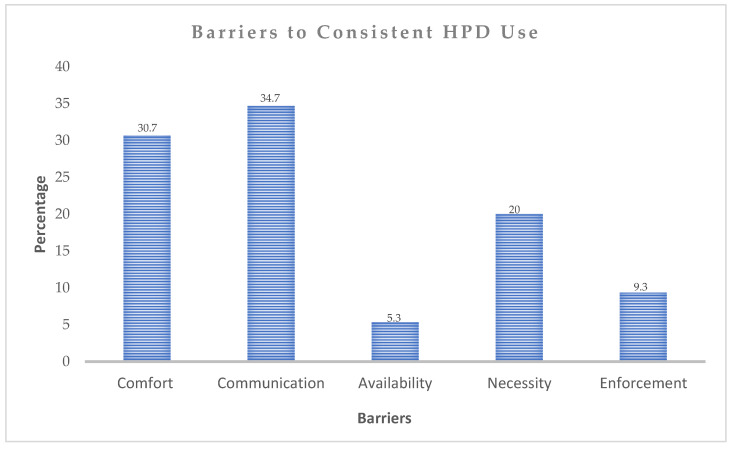
Barriers to consistent HPD use.

Participants who reported sometimes using HPDs were asked to indicate reasons for not using their devices consistently (Figure 4). Several barriers to consistent HPD use were identified, with interference with communication reported by 34.7% of participants, followed by discomfort (30.7%). Limited availability was cited by 5.3% of participants, whereas 20% perceived HPDs as unnecessary, explaining that they were not consistently exposed to noisy environments. In addition, lack of workplace enforcement was reported by 9.3% of participants.

Regardless of the frequency of use, nearly half of the participants (49.6%) reported experiencing some degree of discomfort while using HPDs, whereas 33.9% did not experience discomfort and 16.5% remained neutral. Despite reported discomfort, 90.8% of participants expressed confidence in their ability to use HPDs correctly, whereas 9.2% reported uncertainty. Perceptions regarding the impact of HPDs on communication were mixed: 43.5% reported negative effects, 40% reported no impact, and 16.5% remained neutral.

### 3.3. Workplace Influences and Risk Perception

Awareness of the risks associated with noise-induced hearing loss (NIHL) was high, with 94.8% of participants acknowledging the potential health consequences of occupational noise exposure. Although 64.4% reported having received sufficient training on the correct use of hearing protection devices (HPDs), 28.7% disagreed, with the remaining 6.9% being neutral, indicating variability in training adequacy. Confidence in the effectiveness of HPDs was similarly strong, with 89.6% agreeing that HPDs are effective in preventing hearing loss. Perceived personal vulnerability was also notable, as 60% of participants believed they were at risk of developing hearing loss due to workplace noise, whereas 20% disagreed and a further 20% were unsure.

Despite this high level of awareness and perceived risk, self-reported auditory symptoms were evident among a subset of workers. Just over a quarter (26.4%) reported experiencing ringing in their ears (tinnitus) after their shift, whereas 62.4% did not and 11.1% were uncertain. Similarly, 16.2% of participants reported experiencing temporary hearing loss after work, whereas the majority (75.2%) did not and 8.5% remained neutral. These findings suggest that although knowledge and awareness regarding NIHL and HPD effectiveness was high, a meaningful proportion of workers were already experiencing early auditory symptoms consistent with occupational noise exposure, highlighting the importance of consistent HPD use, effective training, and ongoing hearing conservation measures.

In terms of hearing health monitoring, most participants reported regular engagement in hearing assessments. Only a small proportion (9.4%) reported difficulty hearing conversations in quiet environments, whereas the majority (82.9) did not, and 7.7% were neutral. Regarding formal hearing assessments, 81.9% confirmed that they had undergone a hearing test within the past 12 months, with 15.5% disagreeing and 2.6% remaining neutral. Importantly, the vast majority (88%) reported that they had been informed of their hearing test results, whereas 7.7% had not received their results and 5.1% had not undergone a test at all. These findings indicate that routine hearing monitoring is generally practiced and that most employees are aware of their hearing status, which is critical for the early identification and management of occupational hearing risks.

**Figure 5:**
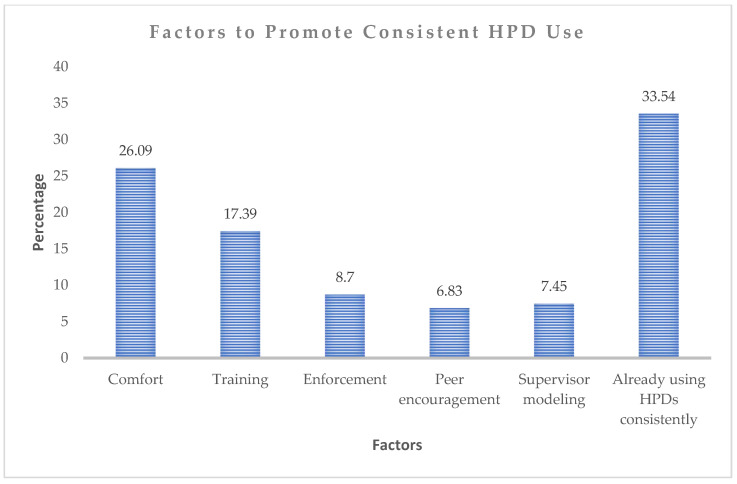
Factors to promote the consistent use of HPDs.

Participants also identified factors that could encourage more consistent use of HPDs (Figure 5). A total of 26.1% indicated that better comfort would motivate them, whereas 17.4% cited more training as a key factor. Improved enforcement of HPD use was noted by 8.7%, peer encouragement by 6.8%, and supervisor modelling by 7.5%. Notably, a substantial proportion of participants (33.5%) reported that nothing would further influence their behaviour, as they already wore HPDs consistently. These findings suggest that comfort, training, and workplace support are important in promoting consistent HPD use, despite a significant portion of workers already adhering to hearing protection practices.

### 3.4. Workplace Safety and Hearing Protection Culture

Participants generally reported a positive perception of occupational health and safety culture in their departments. The majority indicated that occupational health and safety is a priority, and most agreed that hearing protection is an integral part of their department’s culture. Supervisors and colleagues were reported to play a key role in modelling and encouraging HPD use. Many participants agreed that supervisors and colleagues actively encourage HPD use and lead by example. Peer influence was also evident, with a large proportion of participants indicating that seeing other workers wear HPDs influenced their own behaviour. Regular reminders through signs or announcements were commonly reported, suggesting that reinforcement of HPD use is embedded within workplace routines. In addition, most participants noted that the company provides comfortable and effective HPDs, reflecting organizational commitment to supporting a safety-focused culture. These findings suggest a strong hearing protection culture characterized by policy awareness, supervisory modelling, peer influence, and organizational support, though gaps remain in consistent training and addressing comfort-related barriers (Table 2).

### 3.5. Factors Associated with HPD Use Frequency

A series of chi-square tests were conducted to examine associations between HPD use frequency and demographic, perceptual, and workplace factors (Table 3). Participants who reported never using HPDs and age categories with insufficient representation (18–24 years and ≥65 years) were excluded from this analysis.

Age was not significantly associated with HPD use frequency (x^2^(6, *N* = 115) = 4.32, *p* = 0.634). Gender showed a significant association, with males more likely to report consistent HPD use than females (x^2^(4, *N* = 115) = 15.08, *p* = 0.005). Education level was also significantly associated with HPD use frequency (x^2^(6, *N* = 115) = 16.74, *p* = 0.010), with participants who had completed secondary education demonstrating the highest consistency in use. Work experience was not significantly associated with HPD use frequency (x^2^(8, *N* = 115) = 2680, *p* = 0.953).

Perceived discomfort was not associated with HPD use frequency (x^2^(4, *N* = 115) = 3.34, *p* = 0.503). Perceived necessity of HPDs demonstrated a weak trend toward significance (x^2^(2, *N* = 115) = 5.76, *p* = 0.056), but did not reach the threshold for statistical significance.

Confidence in correct HPD use showed a weak-to-moderate association with frequency of use (x^2^(4, *N* = 115) = 9.10, *p* = 0.059; likelihood ratio *p* = 0.022). The perceived effectiveness of HPDs was not associated with use frequency (x^2^(4, *N* = 114) = 3.06, *p* = 0.548); however, confidence in correct use was strongly associated with perceived effectiveness (x^2^(4, *N* = 115) = 17.61, *p* = 0.001).

Type of HPD was significantly associated with consistent use (x^2^(10, *N* = 115) = 74.41, *p* < 0.001), with earmuff users reporting higher adherence than earplug users. The type of noise exposure (x^2^(6, *N* = 115) = 15.27, *p* = 0.018), constant noise exposure (x^2^(6, *N* = 115) = 23.27, *p* < 0.001), and perceived risk of hearing loss (x^2^(6, *N* = 115) = 20.17, *p* = 0.003), were also significantly associated with HPD use frequency.

### 3.6. Logistic Regression Analyses Predicting HPD Use Frequency

Binary logistic regression was conducted to examine associations between demographic, behavioural, and workplace factors and the frequency of hearing protection device (HPD) use (always vs. sometimes). The dependent variable was coded as 0 = always and 1 = sometimes.

#### 3.6.1. Preliminary Model

The first model (Table 4) included demographic and equipment-related variables (age, gender, education level, years of experience, shift length, type of HPD used, confidence in correct HPD use, perceived effectiveness, discomfort, perceived risk of hearing loss, and type of noise). The model was statistically significant (x^2^(11, *N* = 93) = 29.56, *p* = 0.002), indicating that the predictors were significantly associated with HPD use frequency. The model explained 27.2% of the variance according to Cox and Snell R^2^ and 36.3% according to Nagelkerke R^2^. The overall classification accuracy was 72.0%, with 79.6% correct classification for participants who always used HPDs and 63.6% for participants who sometimes used them.

Within this model, education level (B = 1.66, SE = 0.57, Wald = 8.59, *p* = 0.003, OR = 5.26) and type of HPD used (B = 0.42, SE = 0.16, Wald = 6.77, *p* = 0.009, OR = 1.51) were significantly associated with HPD use frequency. None of the remaining variables were significantly associated (*p* > 0.05).

#### 3.6.2. Final Comprehensive Model

A second model included demographic and equipment-related variables plus behavioural and workplace-level factors (forgetting to use HPDs, convenience, pain, confidence in correct use, communication, policy, encouragement, modelling, culture, company reminders, and training). This model was statistically significant (x^2^(22, *N* = 91) = 86.05, *p* < 0.001) and explained 61.2% of the variance according to Cox and Snell R^2^ and 81.7% according to Nagelkerke R^2^. The overall classification accuracy was 92.3%, with 91.8% correct classification for ‘Always’ users and 92.9% for ‘Sometimes’ users.

Significant associations in the final model included:

- Age (B = 6.71, SE = 2.64, Wald = 6.45, *p* = 0.011, OR = 821.69)
- Education level (B = 7.21, SE = 2.71, Wald = 7.06, *p* = 0.008, OR = 1353.82)
- Type of HPD used (B = 1.70, SE = 0.70, Wald = 5.99, *p* = 0.014, OR = 5.49)
- Type of noise (B = 4.38, SE = 1.69, Wald = 6.75, *p* = 0.009, OR = 79.55)
- Forgetting to use HPDs (B = −2.29, SE = 0.92, Wald = 6.12, *p* = 0.013, OR = 0.10)
- Workplace culture (B = 2.81, SE = 1.21, Wald = 5.36, *p* = 0.021, OR = 16.55)
- Training (B = −2.53, SE = 1.12, Wald = 5.11, *p* = 0.024, OR = 0.08)
- Perceived effectiveness of HPDs (B = −7.22, SE = 3.46, Wald = 4.37, *p* = 0.037, OR = 0.001)

All other variables were not significantly associated with HPD use frequency (*p* > 0.05). Interpretation of Large Odds Ratios: Several predictors yielded very large odds ratios, particularly age and education level. These values indicate a strong association and may reflect sparse categories or near-perfect separation between participants who always versus those who sometimes use HPDs. Such ORs should be interpreted in terms of direction and relative strength of the association, rather than as precise magnitudes. For example, participants with higher education were much more likely to sometimes use HPDs compared with lower-educated participants, but this does not imply causation.

### 3.7. Summary of Key Findings

Overall, HPD use was significantly associated with a range of individual, behavioural, and workplace factors. At the individual level, education level, age, the type of HPD used, and the type of noise exposure were significantly associated with HPD use frequency. Behavioural and organizational factors, including forgetting to use HPDs, workplace culture, and training, were also significantly associated with whether HPDs were used consistently. In contrast, gender, work experience, shift length, perceived discomfort, and the perceived effectiveness of HPDs were not significantly associated with HPD use frequency. Collectively, these findings highlight the multifactorial nature of hearing protection behaviour, indicating that consistent HPD use is associated with both personal characteristics and workplace context, rather than any single influencing factor.

## 4. Discussion

This study examined factors associated with the frequency of HPD use among workers exposed to hazardous noise in a South African aluminium manufacturing setting. In keeping with this study’s objectives, we explored associations between demographic characteristics, perceptual factors, device use, noise exposure characteristics, and workplace influences with self-reported HPD use. Our findings indicate that HPD use behaviour is multifactorial, shaped by an interplay of individual characteristics, risk perceptions, device-related factors, noise exposure patterns, and organisational context. Although bivariate analyses identified several significant associations, multivariable logistic regression clarified that only a subset of these variables remained independently associated with consistent HPD use, highlighting the complex interdependencies underlying protective behaviour in occupational environments.

Despite high levels of awareness and access to HPDs in the study population, only half of the participants reported always using HPDs, whereas the remainder reported intermittent or no use. This pattern is consistent with findings from South African industrial contexts, particularly mining, where self-reported use often overestimates actual adherence and may not translate into consistent protective behaviour [28,37,38]. Internationally, similar discrepancies between awareness and behaviour have been observed, highlighting the persistent challenge of converting knowledge into practice [24,33].

Demographically, gender and education level were significantly associated with HPD use frequency in chi-square analyses. Male participants reported higher rates of consistent HPD use than female participants (*p* = 0.005), mirroring studies attributing gender differences to occupational roles, both locally and internationally. Locally, in a cohort of South African gold mine workers, approximately 98% of participants were male and only 0.6% were female, reflecting the male dominance of the mining workforce [39]. Similarly, in a study of a South African steel-making plant, 81.3% of employees were male, further illustrating the predominance of men in similar industrial workplaces [40]. A Brazilian study found significantly higher HPD use among men compared to women, attributing differences to occupational roles and perceptions of risk [32,41]. Tak [42] and Chauhan [43] also reported similar findings. However, in the multivariable model, gender was not an independent predictor of HPD use, suggesting that contextual factors such as workplace culture, enforcement, and role expectations may mediate gendered differences in protective behaviour. This aligns with evidence indicating that strong organisational safety climates can attenuate demographic disparities in safety compliance [24,44].

Education level showed a significant relationship with HPD use in both unadjusted and adjusted analyses, with participants holding secondary education more likely to report consistent use than those with tertiary education. Although higher education is often associated with better safety behaviour in some settings [45,46], South African research has described contexts in which workers with higher formal education perceive lower personal exposure risk or occupy roles with less direct noise exposure, potentially reducing their engagement with protective behaviours [34], whereas in lower-skilled workers, awareness of noise hazards is high; however, consistent HPD use remains sub-optimal [38]. The large odds ratio for education in the regression model may partly reflect sparse category representation rather than a precise effect size, but the association suggests that educational attainment interacts with role expectations and exposure patterns, rather than acting as a simple proxy for knowledge or risk comprehension.

With nearly half of the participants aged 25–44 years and a similar proportion aged 45–64 years, the sample represents a predominantly mature workforce. In this context, age-related auditory changes such as presbycusis, which become more prevalent from mid-adulthood onwards, are relevant when interpreting age-related patterns in HPD use, as they may influence perceived hearing risk and self-reported protective behaviour independent of occupational noise exposure. The limited representation of the youngest (18–24 years) and oldest (≥65 years) age groups further restricts meaningful comparisons across the full age spectrum and warrants caution in interpreting age-related differences.

Statistically, age was not significantly associated with HPD use in the chi-square analysis (*p* = 0.634). However, in the multivariable regression model, age emerged as a significant predictor, indicating that older workers were more likely to report consistent HPD use once behavioural and organisational factors were accounted for. This suggests that the influence of age on HPD use may operate indirectly through factors such as accumulated exposure experience, heightened perceived susceptibility, or personal experience with auditory symptoms. These explanations are consistent with the Health Belief Model, which emphasises perceived susceptibility and severity as key motivators of preventive behaviour [47,48]. Taken together, the observed age-related patterns likely reflect a combination of biological auditory changes and sample characteristics rather than behavioural differences attributable to age alone. Future research incorporating objective audiometric measures would assist in disentangling the relative contributions of occupational noise exposure and age-related hearing loss to HPD use behaviour.

Perceptual factors played a significant role in HPD use. Perceived personal risk of hearing loss was significantly associated with consistent HPD use, aligning with international evidence that higher risk perception is linked to greater adherence to protective behaviours [33,44]. Although awareness of NIHL was high in this sample, nearly one-fifth of participants were uncertain about their personal risk, suggesting that broad awareness does not always translate into personal relevance. Similar findings have been reported by Trimmis, Kaparou [49].

Confidence in correct HPD use was also associated with HPD use frequency at the bivariate level and correlated strongly with perceived effectiveness, reflecting the potential importance of self-efficacy in protective behaviour. Although confidence and perceived effectiveness did not remain independent predictors in the adjusted model, their interrelationship points to the relevance of skills-based and competence-building strategies within hearing conservation programmes [46].

The type of HPD was significantly associated with consistent use, with earmuff users reporting higher adherence than earplug users. This observation is consistent with research identifying comfort, usability, and interference with communication as key determinants of HPD adherence [28,33]. Although discomfort was not statistically significant in inferential tests, its prevalence in descriptive responses indicates that experiential factors remain important barriers. Internationally, situational and interpersonal influences such as comfort, communication interference, and perceived inconvenience have been repeatedly shown to affect HPD use [32,33,50], highlighting the need for interventions that address device usability alongside behavioural messaging. In the South African mining sector, Ntlhakana, Kanji [28] found that comfort, design and communication interference influenced HPD use. The present findings suggest that beyond availability, the suitability of HPDs for the work context, ease of use, and worker preference may be associated with adherence.

Noise exposure characteristics, specifically type and constancy of noise, were significantly associated with HPD use frequency. Workers exposed to more constant noise reported higher consistency of HPD use, possibly reflecting the formation of habitual protective behaviours in predictable exposure contexts. This aligns with evidence that intermittent or variable noise environments can undermine consistent HPD use due to risk normalisation and task prioritisation [24].

One of the study’s most salient findings is the prominence of workplace and organisational factors in predicting HPD use behaviour. The comprehensive multivariable model explained a substantial proportion of variance in HPD use frequency and identified workplace culture, training, and behavioural cues (e.g., forgetting) as significant predictors, even when controlling for demographic and individual factors. This highlights that protective behaviour is not solely an individual choice but is shaped by the broader sociotechnical environment in which work occurs. Workplace safety culture, including supervisory modelling, peer influence, routine reminders, and norms around HPD use, featured strongly in participant perceptions and emerged as an important determinant of consistent use. These findings echo international occupational safety research that positions organisational commitment, visible management support, and social norms as critical drivers of safety behaviour [26,44,51]. In South African industrial settings, where HCPs have often been implemented as discrete elements rather than integrated systems, the role of organisational reinforcement may be particularly important. For example, mining sector studies have noted that audiological input and behavioural reinforcement components are frequently under-emphasised, limiting programme effectiveness [14,52].

Training was significantly associated with HPD use in the regression model, although its effect was complex. Although most participants reported receiving training, a notable proportion perceived it as inadequate. This suggests that the quality, relevance, and practicality of training may be more important than mere exposure to training content, a nuance that has been highlighted in both local and international research [53,54]. Interventions that combine knowledge transfer with hands-on skills training, behavioural rehearsal, and reinforcement may be more effective in enhancing HPD use than didactic training alone.

Behavioural factors such as forgetting to use HPDs also retained significance in the adjusted model, highlighting that non-use is not always intentional resistance but may reflect lapses in habit or attention. Environmental and organisational cues, such as reminders, supervision, and routine practices, may help bridge the gap between intention and action, as evidenced in broader occupational safety literature [24,46,55].

The combined use of chi-square and multivariable logistic regression provided complementary insights into the determinants of HPD use. Several variables that appeared significant in unadjusted analyses did not remain independent predictors in the adjusted model, illustrating that bivariate associations may be confounded or mediated by broader organisational and behavioural factors. Conversely, variables such as age and workplace culture only emerged as significant within the multivariable context, underscoring the importance of considering interactions and conditional effects rather than isolated associations. Together, these findings reinforce the conceptualisation of HPD use as a multilevel behaviour influenced by personal characteristics, perceptual factors, device usability, exposure features, and the organisational environment. This aligns with theoretical frameworks such as the Health Belief Model and social ecological models of occupational safety, which emphasise the nested influence of individual, interpersonal, and organisational determinants on protective behaviour.

### 4.1. Implications for Practice and Policy

The findings highlight several practical and policy implications for hearing conservation in South African workplaces and similar industrial contexts. Organisations should consider providing multiple types of hearing protection devices (HPDs) and actively involving workers in selection and fit-testing processes to improve comfort, usability, and communication compatibility, with earmuffs appearing to support higher adherence in this context.

Training initiatives should extend beyond basic awareness to include correct fitting techniques, comfort management strategies, personalised risk communication, and behavioural reinforcement. Workplace safety culture plays a critical role: supervisors and peers should model and reinforce HPD use consistently, embedding hearing protection into routine safety practices rather than treating it as an individual responsibility.

Importantly, HPD use should be complemented by engineering and administrative controls, including noise reduction at source, zoning, task rotation, and shift design, to reduce reliance on personal protective equipment alone. Observed demographic differences, particularly by gender and education level, suggest that targeted and context-sensitive interventions may be required to address variable compliance. Finally, the provision of HPDs in isolation is insufficient; monitoring actual use, providing feedback, and integrating ongoing audiological input are essential for effective and sustainable hearing conservation programmes.

### 4.2. Limitations and Future Research

This study has several strengths that should be acknowledged. It examined HPD use within a real-world industrial setting, integrating demographic, perceptual, device-related, exposure, and organisational factors within a single analytical framework. The use of both bivariate and multivariable analyses strengthened interpretation by distinguishing independent associations from confounded relationships, whereas the inclusion of locally relevant workplace variables enhances the applicability of the findings to South African occupational health contexts.

Despite these strengths, several limitations warrant consideration. The cross-sectional design precludes causal inference between perceptual, behavioural, and organisational factors and HPD use. The modest sample size may have limited statistical power to detect small or moderate associations, increasing the possibility of Type II error. Although the sample size (*n* = 115) was adequate for exploratory analysis, it represents a single occupational noise environment, which may limit generalisability to other industries or settings. Self-reported HPD use is subject to recall and social desirability bias; future studies could strengthen validity through observational verification or objective monitoring methods. The age distribution was skewed towards mid-adulthood, limiting age-stratified comparisons, and the potential influence of presbycusis on risk perception and HPD use was not directly assessed. Incorporating audiometric testing in future research would help to disentangle age-related hearing loss from occupational noise exposure.

Future research should employ longitudinal designs, include larger and more diverse samples, stratify analyses by HPD type, and explore gender- and education-specific intervention pathways. Qualitative studies examining device comfort, communication trade-offs, and lower adherence among tertiary-educated workers would further enhance our understanding of the contextual barriers to consistent HPD use.

## 5. Conclusions

This study demonstrates that although awareness of occupational noise risk and access to hearing protection devices are high, consistent HPD use among noise-exposed workers remains moderate and is influenced by a complex interplay of demographic, perceptual, device-related, exposure, and organisational factors. These findings are consistent with both South African and international literature and reinforce that effective hearing conservation programmes require more than HPD provision alone.

In the South African context, where occupational noise-induced hearing loss remains a persistent yet under-addressed hazard despite extensive programme implementation, integrating behavioural, device usability, and organisational determinants into hearing conservation programme design offers a pathway toward improved protective behaviour and reduced long-term risk of hearing loss.

## Figures and Tables

**Table 1:** Participant characteristics.

Demographics		Count
Gender	Female	38
	Male	76
	Self-identification	1
Age	18–24	1
	25–44	55
	45–64	58
	65+	1
Education	No formal education	0
	Primary education	2
	Secondary education	75
	Tertiary education	36
	Postgraduate	2
Duration	Less than a year	3
	2–3 years	12
	4–6 years	20
	7–9 years	8
	More than 10 years	72
Shift length	1–3 h	0
	4–6 h	0
	7–11 h	57
	12+ h	58
Occupation	Machine operator	6
	Furnace operator	2
	Maintenance T	2
	Quality control	5
	Forklift driver	31
	Shift leader	1
	Process engineer	1
	Packaging	27
	Cleaning	2
	Administrative	3
	Other	35

**Table 2:** Participant perceptions of workplace safety and hearing protection culture.

Variable	Agree (%)	Neutral (%)	Disagree (%)
Occupational health and safety is a priority	83.7%	6%	10.3%
Hearing protection is part of department culture	79.2%	11.3%	9.5%
Supervisors/colleagues encourage HPD use	82%	7.7%	10.3%
Supervisors model HPD use	70.7%	14.7%	14.6%
Peer behaviour influences HPD use	76.9%	13.7%	9.4%
HPDs provided are comfortable/effective	83%	6.8%	10.2%
Noise hazard zones are clearly labelled	89.5%	3.5%	7%
Workers are reminded to wear HPDs	80.4%	10.2%	9.4%

**Table 3:** Summary of inferential results.

Variables	Chi-Square (χ^2^)	df	*p*-Value	N	Cramér’s V	Significance
Age × Frequency	4.317	6	0.634	115	0.14	Not significant
Gender × Frequency	15.084	4	0.005	115	0.26	Significant
Education level × Frequency	16.738	6	0.010	115	0.27	Significant
Work experience × Frequency	2.680	8	0.953	115	0.11	Not significant
Perceived discomfort × Frequency	16.738	4	0.503	115	0.27	Not significant
Perceived necessity × Frequency	5.756	2	0.56	115	0.22	Weak significance
Perceived effectiveness × Frequency	3.060	4	0.548	115	0.12	Not significant
Correct use × Frequency	9.104	4	0.059	115	0.20	Weak significance
Perceived risk of hearing loss × Frequency	20.168	6	0.003	115	0.30	Significant
Constant noise × Frequency	23.272	6	<0.001	115	0.32	Significant
Type of noise × Frequency	15.274	6	0.018	115	0.26	Significant
Type of HPD × Frequency	7.899	10	0.639	115	0.19	Not significant
Effectiveness × Correct use	17.607	4	0.001	115	0.28	Significant

**Table 4:** Binary Logistic Regression Predicting HPD Use Frequency—Comparison of Preliminary and Final Models.

Predictor	B (Model 1)	SE	Wald	*p*	OR	B (Final Model)	SE	Wald	*p*	OR
Age	0.29	0.50	0.34	0.563	1.34	6.71	2.64	6.45	0.011	821.69
Gender	−1.06	0.57	3.46	0.063	0.35	−1.71	1.39	1.52	0.218	0.18
Education	1.66	0.57	8.59	0.003	5.26	7.21	2.71	7.06	0.008	1353.82
Years of Experience	0.18	0.21	0.74	0.391	1.20	−0.66	0.60	1.21	0.272	0.52
Shift Length	0.04	0.51	0.01	0.935	1.04	0.79	1.28	0.38	0.537	2.20
Type of HPD	0.42	0.16	6.77	0.009	1.51	1.70	0.70	5.99	0.014	5.49
Confidence	1.20	0.80	2.28	0.131	3.33	1.83	1.10	2.76	0.096	6.22
Effectiveness	−0.13	1.31	0.01	0.920	0.88	−7.22	3.46	4.37	0.037	0.001
Uncomfortable	0.48	0.30	2.51	0.113	1.62	0.62	0.86	0.52	0.471	1.86
Perceived Risk	0.21	0.39	0.29	0.587	1.24	−1.41	1.45	0.95	0.331	0.25
Type of Noise	0.90	0.55	2.70	0.101	2.47	4.38	1.69	6.75	0.009	79.55
Occupation	—	—	—	—	—	0.17	0.20	0.74	0.389	1.18
Pain	—	—	—	—	—	0.28	0.71	0.15	0.696	1.32
Forgetting	—	—	—	—	—	−2.29	0.92	6.12	0.013	0.10
Convenience	—	—	—	—	—	−0.92	0.97	0.90	0.342	0.40
Communication	—	—	—	—	—	−1.05	0.70	2.26	0.132	0.35
Policy	—	—	—	—	—	0.29	1.10	0.07	0.790	1.34
Encouragement	—	—	—	—	—	1.84	1.35	1.87	0.172	6.31
Modelling	—	—	—	—	—	0.15	0.88	0.03	0.865	1.16
Culture	—	—	—	—	—	2.81	1.21	5.36	0.021	16.55
Company Reminder	—	—	—	—	—	−1.15	1.25	0.85	0.356	0.32
Training	—	—	—	—	—	−2.53	1.12	5.11	0.024	0.08

Note. The dependent variable was coded as 0 = always and 1 = sometimes. OR = odds ratio. Model 1 = the preliminary model, including demographics and equipment factors. Final Model = the comprehensive model, including demographic, behavioural, and workplace-level variables. Blank entries (—) indicate that the variable was not included in Model 1. Large ORs indicate strong associations, possibly due to sparse categories or near-perfect separation, and should be interpreted as associations rather than causal effects.

## Data Availability

The data presented in this study are available on request from the corresponding author due to privacy restrictions. Approval will be subject to meeting the University’s Ethics processes, as well as signing a Non-Disclosure Agreement.

## Abbreviations

The following abbreviations are used in this manuscript:

ONIHL	Occupational noise-induced hearing loss
HPDs	Hearing Protection Devices
HCPs	Hearing Conservation Programmes
COIDA	Compensation for Occupational Injuries and Disease Act
NIOSH	National Institute for Occupational Safety and Health
PEL	Permissible exposure limit
NIHL	Noise-induced hearing loss
